# Impact of Media Use on Chinese Public Behavior towards Vaccination with the COVID-19 Vaccine: A Latent Profile Analysis

**DOI:** 10.3390/vaccines10101737

**Published:** 2022-10-17

**Authors:** Fangmin Gong, Zhuliu Gong, Zhou Li, Hewei Min, Jinzi Zhang, Xialei Li, Tongtong Fu, Xiaomin Fu, Jingbo He, Zhe Wang, Yujia Wang, Yibo Wu

**Affiliations:** 1 School of Literature and Journalism Communication, Jishou University, Jishou 416000, China; 2 School of Public Health, Peking University, Beijing 100871, China; 3 School of Humanities and Social Sciences, Harbin Medical University, Harbin 150088, China; 4 School of Pharmaceutical Sciences, Shandong University, Jinan 250100, China; 5 Department of Nursing, Shengjing Hospital of China Medical University, Shenyang 110055, China; 6 School of Management, Hainan Medical College, Haikou 571100, China; 7 School of Public Health, North Sichuan Medical College, Nanchong 637100, China; 8 Xiangya School of Public Health, Central South University, Changsha 410017, China

**Keywords:** media use, COVID-19 vaccination, social support, latent profile analysis

## Abstract

(1) Background: research on vaccines has received extensive attention during epidemics. However, few studies have focused on the impact of media use on vaccination behavior and the factors influencing vaccination in groups with different media use degrees; (2) Method: Based on seven items related to media use, a total of 11,031 respondents were categorized by the frequency of media use by using latent profile analysis (LPA). Binary regression analysis was used to study the factors that influence the vaccination behaviors of people with different media use frequencies; (3) Results: All respondents were classified into the following three groups: media use low frequency (9.7%), media use general (67.1%), and media use high frequency (23.2%). Media use low frequency (*β* = −0.608, *p* < 0.001) was negatively associated with COVID-19 vaccination behavior. In the media use low frequency, analysis showed that “aged 41 years or older” *β* = 1.784, *p* < 0.001), had religious belief (*β* = 0.075, *p* < 0.05), were ethnic minorities (*β* = 0.936, *p* < 0.01) and had friends support (*β* = 0.923, *p* < 0.05) were associated with a preference to accept the COVID-19 vaccine. In the media use general, those who aged 41 years old and older (*β* = 1.682, *p* < 0.001), had major depression (*β* = 0.951, *p* < 0.05), had friends support (*β* = 0.048, *p* < 0.001) would be more likely to receive COVID-19 vaccination. However, respondents who live in towns (*β* = −0.300, *p* < 0.01) had lower behaviors to receive vaccination for COVID-19. In the media use high frequency, the respondents who aged 41 or older (*β* = 1.010, *p* < 0.001), were ethnic minorities (*β* = 0.741, *p* < 0.001), had moderate depression (*β* = 1.003, *p* < 0.05) would receive the vaccination for COVID-19 positively; (4) Conclusions: The more occluded the media use is, the less likely the respondents are to get vaccinated against COVID-19. Vaccination behavior is influenced by different factors in groups with different frequencies of media use. Therefore, the government and appropriate departments should make individualized and targeted strategies about COVID-19 vaccination and disseminate the vaccination information to different media use groups.

## 1. Introduction

According to the statistics, more than 551 million people in 221 countries and territories worldwide were infected with COVID-19, resulting in more than 6.34 million deaths until 10 July 2022 [1]. Currently, COVID-19 brings an unprecedented challenge to global public health. It has become the most serious global disaster since World War II [2]. Due to the rapid spread of COVID-19 and a large number of infected people, it is difficult to completely isolate the source of infection. Hence, vaccination is considered a key measure to curb the spread of the COVID-19 epidemic [3,4,5]. Recently, COVID-19 vaccines have been developed and used in many countries [6,7]. Nevertheless, the global effort may cause the public to postpone or refuse to go for COVID-19 vaccination for factors such as the media, public health policy, and vaccine safety [8,9]. For instance, the expected vaccination rates of developed countries such as the United States, France, and Italy are lower than 60%. Meanwhile, vaccination rates of COVID-19 are lower in the Middle East, Russia, Africa, and several European countries [10]. In China, although the majority of the Chinese have been officially confirmed vaccinated, some residents still remain unvaccinated against COVID-19 [11].

Media, the important channel to gain epidemic information, moderates the risk perceptions of the public and affects people’s protective behaviors [12]. Print, broadcast, and social media are considered important factors affecting vaccination behaviors against COVID-19 [13,14,15]. For example, the negative information on social media often causes hesitant behaviors to go for vaccines and reduces the perception of risk, which can lead to public refusal to be vaccinated against COVID-19 [16]. However, it has also been argued that individuals who use new media to obtain information about the pandemic are more likely to be interested in going for COVID-19 vaccination [17].

Although previous studies have focused on factors influencing vaccination behavior, for example, demographic variables, such as age, race, and educational level, and political views [18,19,20], fearfulness, anxiety, stress, depression [21,22,23], and social support [24]. There is no study centered on the relationship of vaccination behavior that employed frequency of media use to divide groups. Additionally, some studies have shown that the public forms the following three categories of groups in the process of accessing information: a group of news avoiders who do not use mass media [25], a group of people who access information through various mass media [26], and a group of people who access information either only through new media or only through traditional media [27]. Significant group heterogeneity exists in the extent of media use by the public. Latent profile analysis (LPA) classifies people into different profiles (i.e., categories) to identify information and seek attributes and patterns [28]. By identifying the different media use categories of people through LPA, we can accurately analyze the related factors that affect the public’s vaccination behavior, so as to achieve accurate communication and improve the vaccination rate of the different media use categories. Therefore, we performed a national survey in 31 provinces of mainland China during the initial and booster vaccination during the COVID-19 period. We used LPA to identify the categories of media use in different groups and used binary regression analysis to analyze the factors that influence vaccination behavior in groups with different frequencies of media use. The results of the study will provide policymakers with scientific and appropriate health propaganda and intervention strategies for future disease epidemics.

## 2. Methods

### 2.1. Research Object

Inclusion criteria: (1) aged 18~60; (2) had the nationality of the People’s Republic of China; (3) China’s permanent resident population with an annual travel time ≤1 month; (4) participated in the study and filled in the informed consent form voluntarily; (5) participants could complete the questionnaire survey by themselves or with the help of investigators; (6) participants could understand the meaning of each item in the questionnaire.

Exclusion criteria: (1) those who are confused or affected by cognitive impairment; (2) those who are participating in other similar research projects; (3) those who are unwilling to cooperate.

If the respondent had the ability to think but did not have enough action ability to answer the questionnaire, the investigator would conduct a one-to-one interview and then answer the questions on his or her behalf. In the process of questionnaire distribution, the principles of research design and statistical requirements were followed to control possible bias in the data collection process. To control the quality of the questionnaires and to ensure that there was no difference between the questionnaires completed on their own and those completed with the help of the investigators, the questionnaires were checked by the investigators before being handed over to the respondents for confirmation. The study subjects were registered and coded. The precautions were re-emphasized to the investigators before the start of the daily survey to ensure that all questionnaires returned by the investigators were available.

The Institutional Review Committee approved the research plan of Jinan University (JNUKY-2021-018). All respondents gave informed consent and volunteered to participate in the survey.

### 2.2. Survey Method

The investigators distributed the questionnaires one-on-one and face-to-face to the public in their respective areas of responsibility with the help of the web-based questionnaire star platform (https://www.wjx.cn/, accessed on 15 September 2021). This survey was conducted from 10 July 2021 to 15 September 2021. Survey respondents responded by clicking on the link. They obtain informed consent from the subject while surveying, and the questionnaire number is entered by the investigator. If the respondent could think but are unable to answer the questionnaire, the investigator will conduct one-on-one questioning and answer instead.

Firstly, the provincial capitals of 23 provinces and 5 autonomous regions of China, 4 municipalities (Beijing, Tianjin, Shanghai, and Chongqing) were directly included, and 2–6 cities were selected from the non-capital prefecture-level administrative regions of each province and autonomous region by using the random number table, making for a total of 120 cities.

Based on the results of the “7th National Population Census in 2021”, surveyors or survey teams (≤10 people) were recruited in these cities to conduct quota sampling (quota attributes are gender, age, and urban-rural distribution) for the 120 urban residents selected, made the samples’ gender, age, and urban-rural distribution, match the demographic characteristics. Each city needed to recruit at least one surveyor or one survey team, with each surveyor responsible for collecting 30–90 questionnaires and each survey team responsible for collecting 100–200 questionnaires.

### 2.3. Research Instruments

#### 2.3.1. Basic Information Survey

The questionnaire covered basic personal information (e.g., gender, age, education level, etc.), media use, social support, and COVID-19 vaccination.

#### 2.3.2. Self-Made Media Usage Scale

The self-made media usage scale was used to measure the type and frequency of respondents’ media usage. After systematically reviewing related books and literature, the research team designed the questionnaire [29,30], and on 7 June, 11 June, 15 June, 18 June, 3 July, and 8 July 2021, experts (all with senior titles and regional representation) were consulted to ensure that the questionnaire is applicable to all media users. Finally, the scale consisted of 7 items, which were used to know the contact frequency of respondents to the following 7 kinds of media: newspapers, magazines, radio, television, books (non-textbooks), personal computers (including tablets), and smartphones. Each item was set with the following 5 options: never use, occasionally use, sometimes use, often use, and almost every day, which were assigned to 1–5 in turn (never use = 1, almost every day = 5). The number of days that the measured person was exposed to various media in one week was used as the scoring basis, and the total score of each option was added as the scoring result, with a total score of 35 points. A higher score indicates that the subjects’ media usage frequency was higher. The Cronbach’s alpha of the scale was 0.70.

#### 2.3.3. Perceived Social Support Scale (PSSS)

The PSSS was used to measure social support [31]. The PSSS consisted of 12 items that assessed the emotional support provided by friends, family, and significant others. There were 7 options for each item, ranging from “extremely disagree” to “extremely agree” which were assigned ratings of 1–7 in turn (significantly disagree = 1, extremely agree = 7). Responses were scored based on the degree of consent for each item. The scores of all items were added together to obtain a score between 12 and 84, which reflected the total degree of social support felt by the individual. The higher the score, the higher the degree of support that one owned. The Cronbach’s alpha of the scale was 0.96.

### 2.4. Statistical Methods

Continuous variables were described as mean ± standard deviation, Chi-square test was used for comparisons between groups, and categorical variables were described as frequencies. We used Mplus 8.3 software to conduct LPA and classify the population types with different media use frequencies according to the media-used seven items. The smaller the value of Akaike information criteria (AIC), Bayesian information criteria (BIC), and adjusted Bayesian information criteria (aBIC), the better the fit of the LPA to the data was. The entropy value was between 0 and 1, and the closer to 1, the more accurate the classification. The significant difference between LMR and BLRT (*p* < 0.05) indicated that the K-type model was superior to the K-1 class model. The number of categories in the model gradually increased from the initial model until the model with the best-fitting data were found. Cardinality tests and binary logistic regression analyses of demographic social factors with other scales and types of media use were performed separately by using SPSS 26.0 software on the basis of retaining the optimal category model. *p* < 0.05 (two-side) is statistically significant.

## 3. Results

### 3.1. LPA of Respondents’ Media Use

We investigated one to six potential profile models, as shown in Table 1. Firstly, from the perspective of model fit, the values of AIC, BIC, and aBIC kept decreasing as the number of categories increased, but the three parameters all showed an increase at five categories, indicating that the Class 5 model did not fit well enough. At the same time, the closer the value of entropy is to 1 in Class 3 and Class 4, and both LMRT and BLRT reached a significant level, indicating a better fit for the model.

Based on the consideration of every indicator, a classification model with three potential categories (C1, C2, and C3) was selected as the classification of the degree of respondents’ media use.

**Figure 1:**
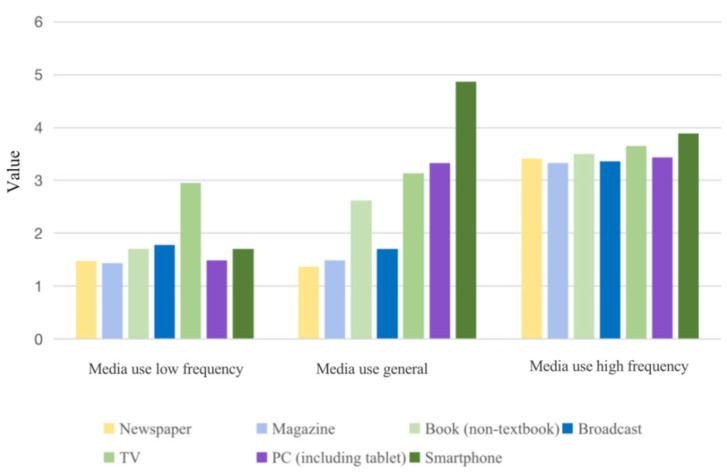
Profile of potential categories of media use.

As shown in Figure 1, three potential categories showed distinct differences in the probability of scoring on the seven items of media use, displaying different characteristics. C1’s score (12.515 ± 1.788) in each item was significantly lower than C2 and C3, accounting for about 9.7% of all subjects. This category was named “media use low frequency” according to the characteristics of their scores; C2 was higher than C1 but lower than C3 in the frequency of media use (18.504 ± 2.643), accounting for about 67.1% of the total subjects, so this category was named “media use general”. The score of C3 (24.571 ± 3.510) was significantly higher than C1 and higher than C2, and this category accounted for about 23.2% of all subjects and was named “media use high frequency”.

### 3.2. Univariate Analysis of Chinese Respondents’ Media Use

As shown in Table 2, a total of 11,031 valid questionnaires were collected in this survey. Among them, 5998 (54.4%) were female, 5332 (48.3%) were aged 19–49, 6487 (58.8%) were educated in a technical college and above, and 8008 (72.6%) were urban residents.

Through the LPA of media use, we found that among the media use low frequency, more respondents were older than 66 years old (35.9%) and married (61.7%). In the media use general, there were more women (57.6%) residents between the ages of 19 and 40 (51.6%). Respondents without depression and anxiety predominated among the three categories of media use. However, in the group of major depression (6.1%), major anxiety (6.4%), and major stress (11.6%), there were more people in the media use high frequency.

The research showed that there was a statistically significant effect of gender, age, religious belief, permanent residence, education level, marital status, monthly per capita household earning, depression, anxiety, and pressure on vaccination (*p* < 0.05), indicating that all these factors were related factors in residents’ behavior towards vaccination.

### 3.3. Media Use and COVID-19 Vaccination Scores of Subjects

The scores of each scale for the included groups were shown in Table 3, in which the total score of the media use scale was (19.34 ± 4.96), the score of newspaper was the lowest (1.86 ± 1.08) and the score of smartphones was the highest (4.33 ± 1.13), indicating that Chinese residents prefer smartphones in terms of media use. The score of COVID-19 vaccination was high (0.89 ± 0.32), indicating that most Chinese residents had been vaccinated against COVID-19.

**Figure 2:**
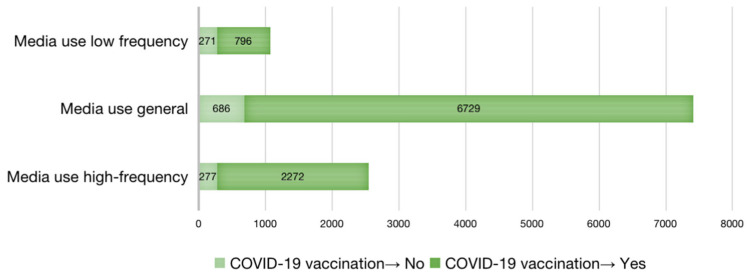
Vaccination statistics for COVID-19 among respondents.

In the summary of respondents’ COVID-19 vaccination scores (Figure 2), 88.8% of them had been vaccinated against COVID-19, and only 11.2% had not been vaccinated against COVID-19.

Among the three groups of media use in COVID-19 vaccination, “media use general” had the highest number of people who had received the vaccine, accounting for 90.7% (6729 persons) of the total number of people who had received the vaccine, followed by “media use high frequency”, accounting for 89.1% (2272 persons). Media use low frequency had the lowest number, accounting for 74.6% (796 persons). Among those who did not receive the COVID-19 vaccine, media use low frequency had the largest number and accounting for 25.4% (271 persons), followed by media use high frequency, accounting for 10.9% (277 persons). Media use general had the lowest number of people and accounting for 9.3% (686 persons).

### 3.4. Binary Logistic Regression Analysis of Factors Affecting COVID-19 Vaccination

In this study, whether the respondents who were vaccinated against the COVID-19 vaccine were used as the dependent variable, the statistically significant variables from the univariate analysis were included as concomitant covariates in a binary logistic regression model for data analysis. The results showed that the behavior of the COVID-19 vaccination in the “media use low frequency” (*β* = −0.608, *p* < 0.001) group was low. The residents who older than 40 years old, (*β* = 1.384, *p* < 0.001), married (*β* = 0.533, *p* = 0.006), had mild depression (β = 0.943, *p* = 0.001), had moderate depression (*β* = 0.920, *p* = 0.001) and had moderate to severe depression (*β* = 1.015, *p* < 0.001) would be more likely to go for COVID-19 vaccination. Instead, the residents who live in the town permanently (*β* = −0.183, *p* = 0.033) would be less likely to receive COVID-19 vaccination. In personal support, friends support (*β* = 0.042, *p* < 0.001) would promote the residents vaccinated against COVID-19. Instead, others’ support (*β* = −0.034, *p* = 0.020) would prevent the residents from receiving the COVID-19 vaccination (Table 4).

We found that the respondents who were aged over 41 years old (*β* = 1.784, *p* < 0.001), had religious belief (*β* = 0.923, *p* = 0.037), and had friends’ support (*β* = 0.923, *p* = 0.011) would receive COVID-19 vaccination (Table 5).

In the “media use general” group, those who were aged over 41 years old (*β* = 1.682, *p* < 0.001), had a monthly household earning per capita in the range of CNY 7501–120,00 (*β* = 0.352, *p* = 0.035), had moderate to severe depression (*β* = 0.951, *p* = 0.033), had friends’ support (*β* = 0.048, *p* = 0.001) would be vaccinated against COVID-19. Instead, the respondents who live in the town permanently would not be vaccinated against COVID-19 (Table 6).

In the “media use high frequency” group, the residents aged over 41(*β* = 1.010, *p* < 0.001), who had moderate depression (*β* = 1.003, *p* = 0.038), would go for COVID-19 vaccination (Table 7).

## 4. Discussion

In this study, the binary regression analysis showed that only the media use low frequency had a significant correlation with vaccination behavior; that is, the more blocked the resident’s media use is, the less likely they would be vaccinated. This is similar to the findings of Antonio Di Mauro et al., (2022). They found that COVID-19 vaccination rates were lower in households that did not receive media interventions compared to households that did [32]. Twitter accounts in vaccine discussions since 2019, which found that up to 45% of people opposed vaccination, while only 24% supported it [33]. The reason may be that the prevalence of conspiracy theories and misinformation in the media has reduced the public’s willingness to be vaccinated against COVID-19 and restricted vaccination behavior [34,35,36]. However, in China, the government has joined forces with all sectors of society and uses official news and social software to convey information on vaccine research and development to the public, which has strengthened residents’ awareness of COVID-19 vaccination [37]. Moreover, the government has also censored The study that is contrary to our findings is Guido’s analysis of the 2,000 most active the content of the media, and a positive tone dominates the Chinese media [38]. In this study, the number of media use high frequency is the largest, followed by the general media use, and the number of media use low frequency is the least, which shows that Chinese residents have more media contact, and most of the media information is positive, which promotes the development of vaccination. Due to the effective use of media by the Chinese government, China is one of the countries with a high rate of COVID-19 vaccination [37]. Malik suggested that in countries with low COVID-19 vaccination rates, the media should disseminate timely and clear information through credible channels to publicize the safety and effectiveness of currently available COVID-19 vaccines and improve vaccination rates [11].

This study also found that in the media use low frequency, the factors affecting the vaccination behavior of COVID-19 were age, social support’s friends support. That is, residents with friend support and over the age of 41 will actively vaccinate against COVID-19, while people aged 19–40 will not. Similar to this finding is the following Japanese study: people aged 20–34 tend to refuse COVID-19 vaccines [39]. Social support was defined as “verbal and nonverbal communication between the recipient and the provider to reduce uncertainty about the situation, self, others, or relationship could help enhance the perception of personal control in life” [40]. Gallagher and other scholars further subdivided social support into the following three categories: family, friends, and other close contacts [41]. In this study, only the friend support dimension of social support had a significant effect on vaccination behavior. In previous studies, it was also confirmed that friend support was an important factor affecting vaccination [42,43].

In addition, the factors that influenced the COVID-19 vaccination behavior in media use were age, depression, and friends’ support. That is, residents aged 41 years or older, with moderate to severe depression, and with friends’ support would actively get the COVID-19 vaccine; the residents whose permanent residence was in town were less likely to get the COVID-19 vaccine. In media use high frequency, minority residents who are older than 41 get vaccinated actively. Age was the influencing factor in vaccination in the three groups, especially for the middle-aged and elderly over 41 years old. Maybe middle-aged and elderly paid more attention to the death risk of COVID-19; they preferred to get vaccinated [44]. It is recommended that the government and related departments pay more attention to the COVID-19 vaccination behaviors of individuals with different levels of media use, particularly those who are occluding the media from their lives. For people with different media usage styles, the government and the appropriate departments must develop individualized and targeted strategies for disseminating information about COVID-19 vaccination to them.

## 5. Highlights and Limitations of Research

This study is the first comprehensive survey and analysis of residents’ attitudes toward COVID-19 vaccination in mainland China, with a large and representative sample size. Additionally, to our knowledge, this is the first study to incorporate media use as an independent variable and to classify the population into potential categories. It represents an innovative approach to the exploration of the extent to which different residents’ media consumption affects their behavior with respect to COVID-19 vaccination.

Aside from the above highlights, this study has the following several additional limitations: first, it is a cross-sectional study, so it can only provide a snapshot of public opinion at the time of the study; second, it relies on cross-sectional data, so analysis of causal inferences is impossible; third, it is based on a web-based questionnaire rather than direct face-to-face interviews or surveys, which may affect the credibility of the collected questionnaires. Fourth, there may be sample selection bias due to the limitations of the sampling method, which may result in some bias in the reporting of their responses. Finally, the research sample of this study is mainly the Chinese public. There is a certain uniqueness in the media used by the Chinese public, so the findings may not match the views of people in other countries.

## 6. Conclusions

During the crisis of the COVID-19 pandemic, the media played an important role in influencing people’s vaccination behaviors. Firstly, this study classified Chinese residents into the following three groups according to the frequency of media use: media use high frequency, general media use, and media use low frequency. Secondly, we investigated the relationship between the types of media use degree of the Chinese residents and their vaccination behavior against COVID-19. We found that the media use occlusion was negatively associated with COVID-19 vaccination behavior in the Chinese region. Finally, we discussed the factors influencing vaccination behavior in groups with different media use degrees and found that age and friend support were important influences on the vaccination of Chinese residents against COVID-19. Firstly, this study provided Chinese samples for the study on vaccination against COVID-19. The classification of LPA provided a brand-new basis for the classification of science popularization targets for public health experts worldwide. Secondly, although this study was conducted in the Chinese region, it provided theoretical and practical significance to the subsequent studies on the underlying mechanism of the influence of media use in the vaccination against COVID-19. Finally, this study was beneficial for the government and relevant departments to develop relative communication strategies, thus enabling precise communication to promote vaccination behavior. Meanwhile, it can also serve as a reference for governments in various countries and regions around the world.

## Figures and Tables

**Table 1:** Potential profile model fit metrics for media use.

Model	K	AIC	BIC	aBIC	Entropy	*p*LMR	*p*BLRT	Class Probability (%)
1	14	246,944.918	247,047.237	247,002.746				1
2	22	230,380.614	230,541.400	230,471.487	0.919	<0.001	<0.001	0.744/0.256
3	30	221,958.644	222,177.898	222,082.562	0.948	<0.001	<0.001	0.097/0.672/0.231
4	38	216,424.795	216,702.517	216,581.758	0.959	<0.001	<0.001	0.089/0.115/0.668/0.128
5	46	208,110.241	208,446.430	208,300.248	0.943	<0.001	<0.001	0.298/0.207/0.262/0.134/0.098
6	54	207,582.155	207,976.812	207,805.207	0.985	0.9944	1.0000	0.449/0.080/0.080/0.239/0.055/0.098

Note. AIC = Akaike information criterion; BIC = Bayesian information criterion; aBIC = adjusted BIC; *p*LMR = *p*-value for LoMendell-Rubin adjusted likelihood ratio test for K vs. K − 1 profiles; *p*BLRT = *p*-value for bootstrapped likelihood ratio test.

**Table 2:** Univariate analysis of Chinese residents’ media use.

Categories	All (N = 11,031, 100.0%)	Media Use Low Frequency (N = 1067, 9.7%)	Media Use General (N = 7415, 67.1%)	Media Use High Frequency (N = 2549, 23.2%)	*χ* *^2^*	*p*
Gender					96.5	*p* < 0.001
Female	5998 (54.4)	538 (50.4)	4268 (57.6)	1192 (46.8)		
Male	5033 (45.6)	529 (49.6)	3147 (42.4)	1357 (53.2)		
Age					1437.2	*p* < 0.001
≤18	1065 (9.7)	109 (10.2)	772 (10.4)	184 (7.2)		
19–40	5332 (48.3)	257 (24.1)	3829 (51.6)	1246 (48.9)		
41–65	3759 (34.1)	318 (29.8)	2570 (34.7)	871 (34.2)		
≥66	875 (7.9)	383 (35.9)	244 (3.3)	248 (9.7)		
Nationality					3.7	*p* = 0.160
The Han nationality	10,386 (94.2)	1001 (93.8)	7003 (94.4)	2382 (93.5)		
Ethnic minorities	645 (5.8)	66 (6.2)	412 (5.6)	167 (6.5)		
r\Religious belief					6.0	*p* = 0.049
Yes	10,709 (97.1)	1035 (97.0)	7181 (96.8)	2493 (97.8)		
No	321 (2.9)	32 (3.0)	233 (3.1)	56 (6.6)		
Permanent residence					217.6	*p* < 0.001
Town	8008 (72.6)	571 (53.5)	5558 (75)	670 (26.3)		
County	3023 (27.4)	496 (46.5)	1857 (25)	1879 (73.7)		
Education level					19.0	*p* = 0.004
Elementary school and above	1127 (10.2))	89 (8.3)	767 (10.3)	271 (10.6)		
Junior middle school	1439 (13.0)	164 (15.4)	984 (13.3)	291 (11.4)		
Technical secondary school/junior high school	1978 (17.9)	185 (17.3)	1360 (18.3)	433 (17.0)		
Junior college and above	6487 (58.8)	629 (59.0)	4304 (58.0)	1554 (61.0)		
Marital status					665.3	*p* < 0.001
Unmarried	4363 (39.6)	263 (24.6)	3115 (42.1)	985 (38.7)		
Married	6226 (56.4)	658 (61.7)	4089 (55.1)	1479 (58.0)		
Divorced	207 (1.9)	14 (1.3)	142 (1.9)	51 (2.0)		
Widowed	235 (2.1)	132 (12.4)	69 (0.9)	34 (1.3)		
Monthly per capita Household earning					214.2	*p* < 0.001
≤3000	3246 (29.4)	486 (45.5)	2099 (28.3)	661 (25.9)		
3001–7500	5325 (48.3)	453 (42.5)	3682 (49.7)	1190 (46.7)		
7501–12,000	1968 (15.4)	84 (7.9)	1166 (15.7)	448 (17.6)		
≥12,001	762 (6.9)	44 (4.1)	468 (6.3)	250 (9.8)		
Whether to have children					1.7	*p* = 0.418
Without	5062 (45.9)	510 (47.8)	3385 (45.7)	1176 (45.8)		
With	5969 (54.1)	557 (52.2)	4030 (54.3)	1382 (54.2)		
Whether to have medical insurance					4.32	*p* = 0.109
Without	2299 (20.8)	224 (21)	1507 (20.3)	568 (22.3)		
With	8732 (79.2)	843 (79)	5908 (79.7)	1981 (77.7)		
Depression					1006.3	*p* < 0.001
No depression	5031 (45.6)	496 (46.5)	3671 (49.5)	864 (33.9)		
Mild depression	3801 (34.5)	384 (36)	2722 (36.7)	695 (27.3)		
Moderate depression	1148 (10.4)	116 (10.9)	672 (9.1)	360 (14.1)		
Moderate to severe Depression	803 (7.3)	56 (5.2)	273 (3.7)	474 (18.6)		
Major depression	248 (2.2)	15 (1.4)	77 (1.0)	156 (6.1)		
Anxiety					982.9	*p* < 0.001
No anxiety	6170 (55.9)	571 (53.5)	4542 (61.3)	1057 (41.4)		
Mild anxiety	3364 (30.5)	358 (33.6)	2324 (31.3)	682 (26.8)		
Moderate anxiety	1198 (10.9)	116 (10.9)	434 (5.9)	648 (25.4)		
Major anxiety	299 (2.7)	22 (2.1)	115 (1.6)	162 (6.4)		
Pressure					282.4	*p* < 0.001
Mild pressure	2719 (24.6)	251 (23.5)	1946 (26.2)	522 (20.5)		
Moderate pressure	7653 (69.4)	704 (66.0)	5217 (70.4)	1732 (67.9)		
Major pressure	659 (6.0)	112 (10.5)	252 (3.4)	295 (11.6)		

**Table 3:** Media use and COVID-19 vaccination scores of subjects.

Categories	Items	The Range of Scores	M ± SD
Newspaper	1	1–5	1.86 ± 1.08
Magazines	1	1–5	1.91 ± 1.05
Books	1	1–5	2.73 ± 1.26
Broadcast	1	1–5	2.10 ± 1.19
Television	1	1–5	3.24 ± 1.28
Personal computer	1	1–5	3.17 ± 1.44
Smart phone	1	1–5	4.33 ± 1.13
COVID-19 vaccination	1	0–1	0.89 ± 0.32

**Table 4:** Binary logistic regression analysis of factors affecting COVID-19 vaccination.

Model		*β*	SE	Wald	*p*	Exp(*β*)	EXP(*β*) 95% Confidence Interval	
							LLCI	ULCI
Independent variable	Media (Ref: General)							
	Occlusion	−0.608	0.099	37.374	<0.001	0.545	0.448	0.662
	High frequency	0.057	0.085	0.450	0.502	1.059	0.896	1.252
Control variable	Gender (Ref: Male)							
	Female	0.033	0.065	0.257	0.612	1.034	0.910	1.174
	Age (Ref: ≤8)							
	19–40	−0.480	0.150	10.256	0.001	0.619	0.461	0.830
	41–65	1.384	0.119	134.877	<0.001	3.992	3.160	5.042
	≥66	1.430	0.110	170.408	<0.001	4.179	3.372	5.180
	Religious belief (Ref: No)							
	Yes	0.303	0.171	3.117	0.077	1.354	0.967	1.894
	Permanent residence (Ref: Rural)							
	Urban	−0.183	0.085	4.568	0.033	0.833	0.705	0.985
	Marital status (Ref: Unmarried)							
	Married	0.533	0.193	7.608	0.006	1.704	1.167	2.488
	Divorced	0.254	0.167	2.314	0.128	1.289	0.929	1.789
	Widowed	0.122	0.286	0.181	0.671	1.130	0.644	1.980
	Per capita monthly household income (Ref: ≤3000)							
	3001–7500	0.184	0.139	1.749	0.186	1.202	0.915	1.579
	7501–12,000	0.249	0.132	3.555	0.059	1.282	0.990	1.660
	≥12,001	0.170	0.149	1.303	0.254	1.185	0.885	1.586
	Education level (Ref: Primary and below)							
	Junior	−0.150	0.107	1.974	0.160	0.860	0.697	1.061
	Secondary, High School	−0.057	0.097	0.345	0.557	0.944	0.781	1.143
	Tertiary and above	−0.138	0.084	2.692	0.101	0.871	0.738	1.027
	Depression (Ref: No depression)							
	Mild depression	0.943	0.289	10.679	0.001	2.568	1.458	4.520
	Moderate depression	0.920	0.282	10.644	0.001	2.510	1.444	4.362
	Moderate to severe depression	1.015	0.280	13.158	<0.001	2.760	1.595	4.778
	Severe depression	0.538	0.267	4.071	0.044	1.713	1.015	2.890
	Anxiety (Ref: No anxiety)							
	Mild anxiety	0.417	0.272	2.345	0.126	1.517	0.890	2.587
	Moderate level	0.257	0.266	0.938	0.333	1.293	0.768	2.176
	Severe anxiety	0.110	0.253	0.190	0.663	1.117	0.679	1.835
	Pressure (Ref: Mild stress)							
	Moderate stress	−0.095	0.150	0.400	0.527	0.910	0.678	1.220
	Severe stress	0.047	0.134	0.124	0.724	1.048	0.806	1.363
	Social support							
	Family support	0.000	0.012	0.001	0.977	1.000	0.976	1.024
	Friends support	0.042	0.012	12.605	< 0.001	1.043	1.019	1.068
	Other support	−0.034	0.015	5.398	0.020	0.967	0.939	0.995

**Table 5:** Binary logistic regression analysis of COVID-19 vaccination in the media use low frequency.

Model	*β*	SE	Wald	*p*	Exp(*β*)	EXP(*β*) 95% Confidence Interval	
						LLCI	ULCI
Age (Ref: ≤ 18)							
19–40	−1.029	0.240	18.380	<0.001	0.357	0.223	0.572
41–65	1.784	0.276	41.715	<0.001	5.954	3.465	10.232
≥ 66	1.629	0.224	53.053	<0.001	5.099	3.289	7.904
Gender (Ref: Male)							
Female	0.073	0.165	0.195	0.659	1.076	0.778	1.486
Religious belief (Ref: No)							
Yes	0.923	0.443	4.348	0.037	2.517	1.057	5.994
Permanent residence (Ref: Rural)							
Urban	−0.066	0.213	0.096	0.757	0.936	0.617	1.421
Marital status (Ref: Unmarried)							
Married	0.096	0.379	0.065	0.799	1.101	0.524	2.316
Divorced	0.069	0.879	0.006	0.937	1.072	0.192	5.999
Widowed	−0.161	0.435	0.136	0.712	0.852	0.363	1.997
Per capita monthly household income (Ref: ≤ 3000)							
3001–7500	−0.226	0.549	0.170	0.680	0.797	0.272	2.339
7501–12,000	−0.094	0.545	0.030	0.862	0.910	0.313	2.646
≥ 12,001	−0.315	0.600	0.276	0.599	0.730	0.225	2.363
Education level (Ref: Primary and below)							
Junior	−0.129	0.301	0.184	0.668	0.879	0.487	1.587
Secondary, High School	−0.178	0.230	0.603	0.437	0.837	0.533	1.312
Tertiary and above	−0.114	0.213	0.288	0.592	0.892	0.588	1.354
Depression (Ref: No depression)							
Mild depression	−0.261	0.969	0.073	0.787	0.770	0.115	5.145
Moderate depression	−0.421	0.964	0.191	0.662	0.656	0.099	4.343
Moderate to severe depression	−0.337	0.973	0.120	0.729	0.714	0.106	4.811
Severe depression	−1.164	0.953	1.492	0.222	0.312	0.048	2.021
Anxiety (Ref: No anxiety)							
Mild anxiety	1.172	0.774	2.291	0.130	3.229	0.708	14.732
Moderate level	0.581	0.766	0.575	0.448	1.788	0.398	8.026
Severe anxiety	0.898	0.760	1.397	0.237	2.454	0.554	10.876
Pressure (Ref: Mild stress)							
Moderate stress	−0.345	0.368	0.880	0.348	0.708	0.344	1.457
Severe stress	−0.064	0.324	0.039	0.843	0.938	0.497	1.770
Social support							
Family support	−0.038	0.032	1.424	0.233	0.962	0.903	1.025
Friends support	0.075	0.029	6.422	0.011	1.077	1.017	1.142
Other support	−0.051	0.037	1.927	0.165	0.950	0.883	1.021

**Table 6:** Binary logistic regression analysis of COVID-19 vaccination in the media use general.

Model	*β*	SE	Wald	*p*	Exp(*β*)	EXP(*β*) 95% Confidence Interval	
						LLCI	ULCI
Age (Ref: ≤18)							
19–40	−0.251	0.216	1.352	0.245	0.778	0.510	1.188
41–65	1.682	0.180	87.020	<0.001	5.379	3.777	7.659
≥66	1.844	0.172	115.282	<0.001	6.325	4.517	8.857
Gender (Ref: Male)							
Female	0.107	0.086	1.535	0.215	1.113	0.940	1.318
Religious belief (Ref: No)							
Yes	0.110	0.226	0.234	0.628	1.116	0.716	1.738
Permanent residence (Ref: No)							
Urban	−0.300	0.115	6.863	0.009	0.741	0.592	0.927
Marital status (Ref: Unmarried)							
Married	0.679	0.349	3.774	0.052	1.972	0.994	3.911
Divorced	0.279	0.325	0.735	0.391	1.322	0.699	2.502
Widowed	0.253	0.455	0.308	0.579	1.287	0.527	3.144
Per capita monthly household income (Ref: ≤3000)							
3001–7500	0.349	0.179	3.793	0.051	1.417	0.998	2.013
7501–12,000	0.352	0.167	4.438	0.035	1.422	1.025	1.973
≥12,001	0.463	0.191	5.880	0.015	1.589	1.093	2.312
Education level (Ref: Primary and below)							
Junior	−0.151	0.137	1.208	0.272	0.860	0.657	1.126
Secondary, High School	0.008	0.128	0.004	0.952	1.008	0.784	1.296
Tertiary and above	−0.091	0.110	0.673	0.412	0.913	0.735	1.134
Depression (Ref: No depression)							
Mild depression	0.895	0.455	3.874	0.049	2.447	1.004	5.966
Moderate depression	0.794	0.446	3.165	0.075	2.211	0.923	5.300
Moderate to severe depression	0.951	0.446	4.547	0.033	2.587	1.080	6.199
Severe depression	0.648	0.445	2.128	0.145	1.913	0.800	4.572
Anxiety (Ref: No anxiety)							
Mild anxiety	−0.099	0.429	0.053	0.818	0.906	0.391	2.099
Moderate level	−0.093	0.420	0.049	0.825	0.911	0.400	2.077
Severe anxiety	−0.186	0.413	0.204	0.652	0.830	0.370	1.864
Pressure (Ref: Mild stress)							
Moderate stress	−0.086	0.246	0.121	0.728	0.918	0.566	1.488
Severe stress	0.076	0.232	0.107	0.744	1.079	0.685	1.700
Social support							
Family support	0.020	0.015	1.717	0.190	1.020	0.990	1.050
Friends support	0.048	0.015	10.256	0.001	1.049	1.019	1.080
Other support	−0.027	0.018	2.189	0.139	0.973	0.939	1.009

**Table 7:** Binary logistic regression analysis of COVID-19 vaccination in the media use high frequency.

Model	*β*	SE	Wald	*p*	Exp(*β*)	EXP(*β*) 95% Confidence Interval	
						LLCI	ULCI
Age (Ref: ≤18)							
19–40	−0.256	0.301	0.724	0.395	0.774	0.430	1.396
41–65	1.010	0.221	2.947	<0.001	2.746	1.782	4.233
≥66	0.841	0.212	15.779	<0.001	2.319	1.531	3.512
Gender (Ref: male)							
Female	−0.150	0.134	1.268	0.260	0.860	0.662	1.118
Religious belief (Ref: No)							
Yes	0.521	0.384	1.844	0.174	1.683	0.794	3.570
Permanent residence (Ref: county)							
Town	−0.040	0.175	0.051	0.821	0.961	0.682	1.354
Marital status (Ref: unmarried)							
Married	0.137	0.506	0.073	0.786	1.147	0.425	3.094
Divorced	0.034	0.487	0.005	0.944	1.035	0.399	2.686
Widowed	−0.383	0.615	0.388	0.534	0.682	0.204	2.275
Monthly per capita household earning (Ref: ≤3000)							
3001–7500	0.065	0.257	0.063	0.801	1.067	0.645	1.764
7501–12,000	0.136	0.239	0.322	0.570	1.146	0.716	1.832
≥12,001	−0.216	0.264	0.669	0.413	0.806	0.481	1.351
Highest education level (Ref: elementary school and below)							
Junior middle school	−0.106	0.219	0.235	0.628	0.899	0.585	1.382
Technical secondary school/junior high school	−0.073	0.215	0.115	0.734	0.930	0.610	1.417
Technical college and above	−0.217	0.172	1.576	0.209	0.805	0.574	1.129
Depression (Ref: depression)							
Mild depression	0.573	0.506	1.283	0.257	1.773	0.658	4.776
Moderate depression	1.003	0.484	4.288	0.038	2.726	1.055	7.041
Moderate to severe depression	0.888	0.462	3.703	0.054	2.431	0.984	6.007
Major depression	0.457	0.425	1.153	0.283	1.579	0.686	3.631
Anxiety (Ref: no anxiety)							
Mild anxiety	0.715	0.492	2.107	0.147	2.044	0.778	5.366
Mild anxiety	0.270	0.469	0.331	0.565	1.310	0.522	3.286
Major anxiety	0.040	0.425	0.009	0.925	1.041	0.453	2.394
Pressure (Ref: mild pressure)							
Moderate pressure	0.190	0.254	0.557	0.455	1.209	0.735	1.988
Major pressure	0.127	0.196	0.418	0.518	1.135	0.773	1.668
Social support							
Family support	0.000	0.032	0.000	1.000	1.000	0.939	1.064
Friends support	0.000	0.031	0.000	0.992	1.000	0.942	1.062
Others’ support	−0.027	0.036	0.570	0.450	0.973	0.906	1.045

## Data Availability

Data are available, upon reasonable request, by emailing: bjmuwuyibo@outlook.com.
